# Presence of Fibroids on Transvaginal Ultrasonography in a Community-Based, Diverse Cohort of 996 Reproductive-Age Female Participants

**DOI:** 10.1001/jamanetworkopen.2023.12701

**Published:** 2023-05-10

**Authors:** David Huang, Brady Magaoay, Mitchell P. Rosen, Marcelle I. Cedars

**Affiliations:** 1 Department of Obstetrics, Gynecology & Reproductive Sciences, University of California, San Francisco

## Abstract

**Importance:**

Fibroids are benign uterine tumors that can cause significant morbidity. Knowledge on fibroid prevalence, especially in the asymptomatic population and in Asian and Hispanic or Latina individuals, is limited, and a better understanding of affected groups will improve timely diagnosis and motivate appropriate recruitment in clinical trials to reduce health disparities.

**Objective:**

To estimate the prevalence of fibroids in a diverse cohort of female individuals.

**Design, Setting, and Participants:**

This cross-sectional study included female individuals not seeking treatment for fertility or other conditions who were prospectively recruited in an academic medical center in San Francisco, California. Effort was made to recruit an equal proportion of participants from 4 large racial and ethnic groups in the United States (Asian-Chinese, Black or African American, Hispanic or Latina, and White) and across 4 equal age groups between 25 and 45 years. All participants reported regular menses (21-35 days), had not used estrogen- or progestin-containing medications in the 3 months prior to enrollment, and denied history of pelvic surgery. The assessment of ultrasonography results was part of an ongoing longitudinal cohort, with initial recruitment from October 2006 to September 2012. Data analysis was performed in April to September 2022.

**Main Outcomes and Measures:**

Fibroid presence and burden as assessed by transvaginal ultrasonography.

**Results:**

A total of 996 female participants were included in the analysis, including 229 (23.0%) Asian-Chinese, 249 (25.0%) Black or African American, 237 (23.8%) Hispanic or Latina, and 281 (28.2%) White individuals. Mean (SD) age was 34.8 (5.7) years in Asian-Chinese participants, 35.4 (6.1) years in Black or African American participants, 34.8 (5.3) years in Hispanic or Latina participants, and 35.3 (5.0) years in White participants. Fibroids were present in 21.8% (95% CI, 16.7%-27.8%) of Asian-Chinese participants, 35.7% (95% CI, 29.8%-42.0%) of Black or African American participants, 12.7% (95% CI, 8.7%-17.6%) of Hispanic or Latina participants, and 10.7% (95% CI, 7.3%-14.9%) of White participants. Black or African American and Asian-Chinese participants were more likely to have fibroids than White participants (Black or African American: adjusted odds ratio [OR], 4.72 [95% CI, 2.72-8.18]; *P* < .001; Asian-Chinese: adjusted OR, 3.35 [95% CI, 1.95-5.76]; *P* < .001). In those with fibroids, the proportion with multiple fibroids were 48.3% in Black or African American participants, 33.3% in White participants, 33.3% in Hispanic or Latina participants, and 26.0% in Asian-Chinese participants (*P* = .06). The largest mean (SD) fibroid diameter was 3.9 (1.9) cm in Black or African American participants, 3.2 (1.6) cm in Asian-Chinese participants, 3.2 (1.6) cm in White participants, and 3.0 (1.4) cm in Hispanic or Latina participants (*P* = .03).

**Conclusions and Relevance:**

In this study of female participants in a nonclinical setting, Black or African American and Asian-Chinese participants were disproportionately affected by uterine fibroids. Hispanic or Latina participants had similar fibroid burden to White participants.

## Key Points

**Question:**

What proportion of reproductive-age female individuals in a nonclinical setting carry uterine fibroids, and which racial and ethnic groups are most affected?

**Findings:**

In this cross-sectional study of 996 reproductive-age female participants, the overall prevalence of fibroids was 20%. Black or African American (35.7%) and Asian-Chinese (21.8%) participants were disproportionately affected compared with White (10.7%) and Hispanic (12.7%) participants.

**Meaning:**

These findings suggest that health care professionals should be cognizant of the high prevalence of uterine fibroids, especially in Black or African American and Asian-Chinese groups, to facilitate timely diagnosis and reduce health disparities associated with this pathology.

## Teaser

This cross-sectional study estimates the prevalence of fibroids in female participants from the 4 largest racial and ethnic groups in the United States.

## Introduction

Uterine fibroids, also known as leiomyomas, are the most common neoplasm affecting female individuals.^1,2^ They are composed of monoclonal smooth muscle cells and fibrous connective tissue, and vary widely in size, location, and growth patterns among individuals. While noncancerous, fibroids can cause significant morbidity. Female individuals with fibroids may experience heavy menstrual bleeding resulting in anemia, as well as pelvic bulk symptoms and pain, thereby prompting frequent outpatient visits, emergency department evaluations, and hospitalizations that often lead to invasive surgical treatments.^3,4^ It is estimated that approximately 30 000 myomectomies are done annually in the United States, and 37% of hysterectomies for benign indications are attributed to fibroids.^5,6^ When considering both direct and indirect costs, fibroids were estimated to cost the United States $5.9 to $34.4 billion annually.^6^

Uterine fibroids also affect critical aspects of a person’s reproductive health. For instance, fibroids are associated with subfertility and early pregnancy loss, possibly through uterine cavity distortion as well as dysregulation of key players in uterine development and receptivity for embryo implantation.^7,8,9,10^ Furthermore, fibroids negatively affect obstetrical outcomes. Pregnant individuals with fibroids are at an increased risk for fetal malpresentation, preterm delivery, abnormal placentation, placental abruption, and worse neonatal outcomes.^11,12,13^ Uterine fibroids should therefore be considered a serious public health issue that deserves the attention of all health care professionals.

Prevalence of uterine fibroids, especially in Hispanic or Latina and Asian female individuals in the United States, remains largely unknown. It is also unclear how prevalent fibroids are in young, asymptomatic female individuals. Current estimates of fibroid prevalence largely stem from studies that evaluated White and Black participants who reported symptoms or underwent surgical treatment.^14,15,16,17,18^ Fibroid presence can also be underestimated, particularly in nonclinical populations, without the use of transvaginal ultrasonography.^19^ In prior literature, it was well-characterized that Black female individuals are disproportionately affected by this pathology in terms of prevalence, burden, and earlier age of onset.^20,21,22^ Compounded by systemic racism and inequities in access to care, Black patients typically present with greater disease severity that likely contributes to worse surgical outcomes and higher complication rates.^14,23,24^ To address the major knowledge gap on fibroid prevalence and distribution in the United States, our study aimed to estimate the proportion of individuals with leiomyomas using a diverse, nonclinical, community-based cohort of reproductive-age female individuals.

## Methods

### Patient Cohort

A total of 996 female individuals aged 25 to 45 years who were not seeking treatment for fertility or other medical conditions were prospectively enrolled in a community-based cohort from October 2006 to September 2012. This population was derived from the University of California San Francisco Ovarian Aging (OVA) study, which is an ongoing longitudinal cohort designed to observe and characterize reproductive aging. Effort was made to recruit a fairly equal proportion of participants based on self-identification as 1 of the 4 large racial and ethnic groups in the United States (Asian-Chinse, Black or African American, Hispanic or Latina, and White). Ethnicity determination required both parents to be of the identified racial and ethnic group. Additionally, recruitment efforts enrolled fairly equal numbers in each of the 4 age categories (25-29 years; 30-34 years; 35-39 years; and 40-45 years). All participants reported regular menses, had not used estrogen- or progestin-containing medications in the 3 months prior to enrollment, and denied a history of ovarian or uterine surgery. We did not exclude patients based on a prior diagnosis of fibroids. Further details of the OVA cohort, study design, and methods have been previously published.^25,26,27^ Written informed consent was obtained from study participants, and institutional review board approval was obtained from the University of California, San Francisco. The Strengthening the Reporting of Observational Studies in Epidemiology (STROBE) reporting guidelines were followed for this study.

### Primary Outcome and Measures

Cross-sectional assessment of fibroid presence was systematically determined for all patients at time of initial examination. All participants underwent a transvaginal ultrasonography performed by 2 board-certified reproductive endocrinologists (M.P.R and M.I.C.) to evaluate for any ovarian or uterine lesions. Transvaginal ultrasonography is a highly sensitive and specific method in ascertaining fibroid burden.^28^ Presence, multiplicity, and dimensions of uterine fibroids were documented. Demographic information, including race and ethnicity, smoking status, parity, and highest level of educational attainment, was reported by participants using a standardized survey. Anthropometric measurements were obtained at time of initial examination. Data analysis regarding fibroid presence was performed in April to September 2022.

### Statistical Analysis

Participant demographic characteristics were described and compared among racial and ethnic groups using analysis of variance (ANOVA) and the χ^2^ test as appropriate. Parameters of fibroid burden were compared using χ^2^ test and ANOVA as appropriate. Univariable logistic regression was used to evaluate the association between race and ethnicity and fibroid presence. Multivariable analysis was performed to evaluate the association between race and ethnicity and fibroid presence, adjusted by age (model 1). An exploratory multivariable analysis was also performed to adjust for additional covariates previously reported to be associated with fibroid prevalence, including prior parity, current smoking, body mass index (BMI; calculated as weight in kilograms divided by height in meters squared) of 30 or greater, and education level (model 2). Estimates of covariates used in the multivariable analysis models are provided in the eTable in Supplement 1. The margins command (Stata version 17.0) was used to provide estimates of the average probability of fibroid presence for levels of interactions between race and ethnicity groups and age categories. The average probabilities were adjusted according to the multivariable logistic regression analysis used in model 2, described previously. All statistical tests were 2-tailed and performed at the .05 level of significance in Stata version 17.0 (StataCorp) without correction for multiple comparisons. Multivariable analyses results should therefore be interpreted as exploratory.

## Results

### Patient Demographic Characteristics

A total of 996 participants were included in the analysis, which consisted of 229 (23.0%) Asian-Chinese, 249 (25.0%) Black or African American, 237 (23.8%) Hispanic or Latina, and 281 (28.2%) White participants. Demographic information is shown in Table 1, stratified by race and ethnicity. The mean (SD) age of the cohort was 35.1 (5.5) years. Mean (SD) age was 34.8 (5.7) years in Asian-Chinese, 35.4 (6.1) years in Black or African American, 34.8 (5.3) years in Hispanic or Latina, and 35.3 (5.0) years in White participants. The racial and ethnic groups exhibited differences in other demographic parameters. Black or African American participants had the highest BMI compared with other racial and ethnic groups. White participants had the highest rate of participants who endorsed current smoking (37 [13.2%]). Most White (240 [85.4%]) and Asian-Chinese (141 [61.6%]) participants were nulliparous at the time of examination. The percentage of participants who completed college education was lower among Hispanic/Latina participants (26.2% [62 participants]).

**Table 1.** Baseline Demographic Characteristics by Race and Ethnicity

Characteristic	Participants, No. (%)				
	White	Black	Hispanic	Asian	All
Total	281 (28.2)	249 (25.0)	237 (23.8)	229 (23.0)	996 (100)
Age, y					
25-29	48 (17.1)	61 (24.5)	52 (21.9)	62 (27.1)	223 (22.4)
30-34	88 (31.3)	49 (19.7)	66 (27.9)	52 (22.7)	255 (25.6)
35-39	93 (33.1)	59 (23.7)	70 (29.5)	59 (25.8)	281 (28.2)
40-45	52 (18.5)	80 (32.1)	49 (20.7)	56 (24.4)	237 (23.8)
Age, mean (SD), y	35.3 (5.0)	35.4 (6.1)	34.8 (5.3)	34.8 (5.7)	35.1 (5.5)
BMI, mean (SD) [range]^a^	24.5 (5.5) [17.4-58.4]	32.1 (8.0) [16.5-54.9]	29.6 (6.4) [15.8-53.1]	23.0 (3.6) [17.2-43.7]	27.3 (7.1) [15.8-58.4]
Current Smoking^b^					
Yes	37 (13.2)	26 (10.4)	12 (5.1)	6 (2.6)	81 (8.1)
No	244 (86.8)	223 (89.6)	225 (94.9)	223 (97.4)	915 (91.9)
Parity status^b^					
Nulliparous	240 (85.4)	113 (45.4)	73 (30.8)	141 (61.6)	567 (56.9)
Parous	41 (14.6)	136 (54.6)	164 (69.2)	88 (38.4)	429 (43.1)
Education^b^					
High school	7 (2.5)	41 (16.5)	107 (45.1)	32 (14.0)	187 (18.8)
Some college	35 (12.5)	108 (43.4)	68 (28.7)	37 (16.2)	248 (24.9)
College	138 (49.1)	67 (26.9)	44 (18.6)	107 (46.7)	356 (35.7)
Graduate or professional	101 (35.9)	33 (13.3)	18 (7.6)	52 (23.1)	205 (20.6)

Abbreviation: BMI, body mass index (calculated as weight in kilograms divided by height in meters squared).

^a^ One-way analysis of variance, *P* < .001.

^b^ χ^2^ test, *P* < .001.

### Fibroid Presence and Characteristics

**Figure 1.**
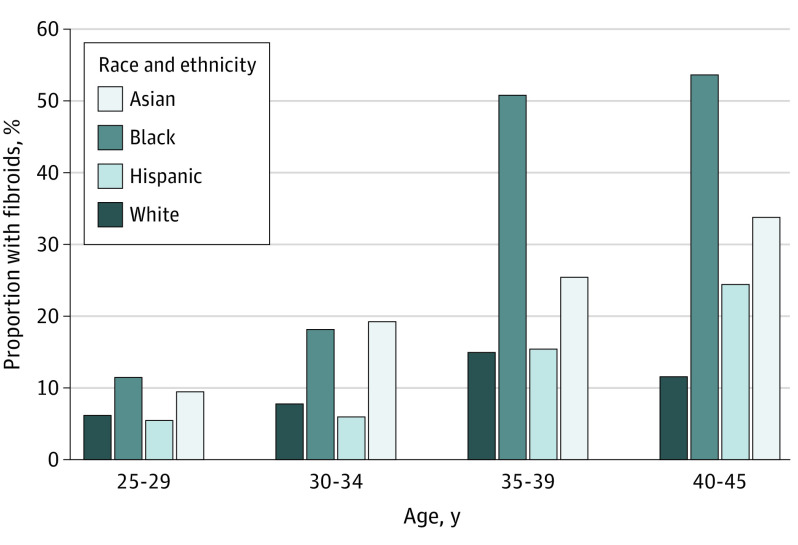
Proportion of Participants With Fibroids by Age and Race and Ethnicity

Fibroid presence, multiplicity, and size were assessed using transvaginal ultrasonography and compared among racial and ethnic groups (Table 2). Overall, fibroids were present in 20.0% of participants in our cohort. When analyzed by racial and ethnic groups, fibroids were present in 21.8% (95% CI, 16.7%-27.8%) of Asian-Chinese, 35.7% (95% CI, 29.8%-42.0%) of Black or African American, 12.7% (95% CI, 8.7%-17.6%) of Hispanic or Latina, and 10.7% (95% CI, 7.3%-14.9%) of White participants (*P* < .001). Fibroid prevalence increased with age, with Black or African American and Asian-Chinese participants disproportionately affected by fibroids in all age groups (Figure 1). Increased fibroid prevalence with age was also observed in Hispanic or Latina participants. On the contrary, this finding was not observed in White participants in this study. In those with fibroids, the differences in proportion of participants with multiple fibroids were not statistically significant: 48.3% in Black or African American, 33.3% in White, 33.3% in Hispanic or Latina, and 26.0% in Asian-Chinese participants (*P* = .06). The largest mean (SD) diameter of fibroids was 3.9 (1.9) cm in Black or African American participants, followed by 3.2 (1.6) cm in Asian-Chinese, 3.2 (1.6) cm in White, and 3.0 (1.4) cm in Hispanic or Latina participants (*P* = .03). Pairwise comparisons showed that this variance was attributed to the fibroid size difference between Black or African American and Hispanic or Latina participants (mean [SD] diameter, 3.9 [1.9] cm vs 3.0 [1.4] cm; *P* = .01), as well as between Black or African American and Asian-Chinese participants (mean [SD] diameter, 3.9 [1.9] cm vs 3.2 [1.6] cm; *P* = .03).

**Table 2.** Fibroid Characteristics by Race and Ethnicity

Characteristic	Participants, No. (%)				
	White	Black	Hispanic	Asian	All
Total	281 (28.2)	249 (25.0)	237 (23.8)	229 (23.0)	996 (100.0)
Presence of fibroids^a^					
Yes	30 (10.7)	89 (35.7)	30 (12.7)	50 (21.8)	199 (20.0)
No	251 (89.3)	160 (64.3)	207 (87.3)	179 (78.2)	797 (80.0)
Solitary vs multiple^b^					
Solitary	19 (63.3)	46 (51.7)	20 (66.7)	37 (74.0)	122 (61.6)
Multiple	10 (33.3)	43 (48.3)	10 (33.3)	13 (26.0)	76 (38.4)
Missing data	1 (3.3)	0	0	0	0
Largest dimension, mean (SD) [range], cm^c^	3.2 (1.6) [1.3-8.0]	3.9 (1.9) [1.3-10.6]	3.0 (1.4) [1.2-6.5]	3.2 (1.6) [0.9-7.9]	3.5 (1.7) [0.9-10.6]

^a^ χ^2^ test, *P* < .001.

^b^ χ^2^ test, *P* = .06.

^c^ One-way analysis of variance, *P* = .03.

### Association Between Race and Ethnicity and Uterine Fibroids

**Figure 2.**
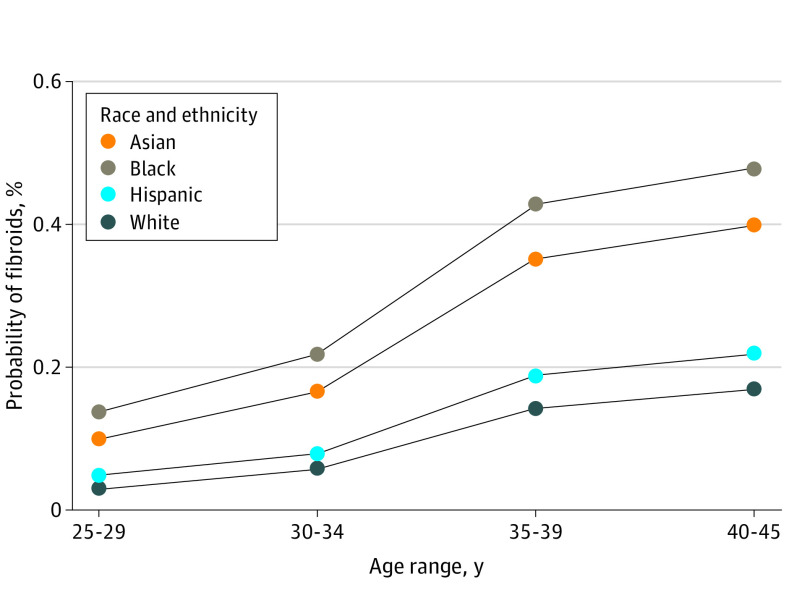
Probability of Fibroid Presence Adjusted probabilities of fibroid presence by age category and race and ethnicity.

Univariable logistic regression analyses identified Black or African American and Asian-Chinese race and ethnicity as strongly associated with presence of uterine fibroids (Table 3). Multivariable logistic regression analyses, adjusted for age as well as additional covariates postulated to be associated with fibroid prevalence, was then performed (Table 3). Black or African American and Asian-Chinese race and ethnicity remained independently associated with uterine fibroids; age and race and ethnicity most affected the odds of having fibroids (Table 3 and the eTable in Supplement 1). Age older than 35 years, compared with age younger than 30 years, was associated with increased odds of uterine fibroids (age 35-39 years: adjusted odds ratio [OR], 5.01 [95% CI, 2.80-8.95]; *P* < .001; age 40-45 years: adjusted OR, 6.18 [95% CI, 3.46-11.05]; *P* < .001). Compared with White participants, Black or African American and Asian-Chinese participants were more likely to have fibroids (Black or African American: adjusted OR, 4.72 [95% CI, 2.72-8.18]; *P* < .001; Asian-Chinese participants: adjusted OR, 3.35 [95% CI, 1.95-5.76]; *P* < .001). Using data from this cohort and the multivariable logistic regression model described previously, we generated a plot with probabilities of fibroid presence by age group in all 4 large racial and ethnic groups in the United States (Figure 2).

**Table 3.** Association Between Race and Ethnicity and Presence of Fibroids

Variable	Unadjusted		Adjusted			
			Model 1^a^		Model 2^b^	
	OR (95% CI)	*P* value	OR (95% CI)	*P* value	OR (95% CI)	*P* value
Race and ethnicity (compared with White)						
Black	4.65 (2.94-7.36)	<.001	4.85 (3.01-7.81)	<.001	4.72 (2.72-8.18)	<.001
Hispanic	1.21 (0.71-2.08)	.48	1.24 (0.72-2.15)	.43	1.40 (0.74-2.64)	.31
Asian	2.34 (1.43-3.82)	.001	2.50 (1.51-4.54)	<.001	3.35 (1.95-5.76)	<.001

Abbreviation: OR, odds ratio.

^a^ Model 1 was adjusted for age.

^b^ Model 2 was adjusted for age, prior parity, current smoking, obesity (body mass index [calculated as weight in kilograms divided by height in meters squared] ≥30), and college education attainment.

## Discussion

Prior knowledge on fibroid prevalence and associated health disparities focused primarily on symptomatic White and Black participants. Here, we provide estimates on the proportion of participants in the US San Francisco Bay Area with uterine fibroids in a nonclinical setting by prospectively examining a diverse, community-based cohort of 996 reproductive-age female individuals.

Older age and race and ethnicity were most significantly associated with increased odds of fibroid diagnosis on ultrasonography. Consistent with prior literature in both asymptomatic and symptomatic populations, Black or African American participants tended to carry larger, more numerous fibroids.^1,2,14,29^ In this community-based cohort, Asian-Chinese participants were also disproportionately affected by fibroids compared with White participants, which has not, to our knowledge, been previously reported. Prior data for fibroid prevalence in Hispanic or Latina participants are also limited. Here, we found that Hispanic or Latina participants exhibited similar fibroid burden as White participants in a nonclinical setting. The higher proportion of participants with uterine fibroids was observed in the Black or African American and Asian-Chinese cohorts across all age groups (Figure 2), suggesting some shared underlying risk factor(s) in these groups. Given our large sample size, we performed an exploratory multivariable regression analysis with additional sociodemographic factors. Findings from this exploratory analysis also suggested obesity and current smoking as independent risk factors for fibroid presence (eTable in Supplement 1).

Our study adds to prior hypotheses and findings regarding the pathophysiology of fibroids. Fibroid growth is hormonally responsive and thought to be predominantly driven by estrogen. Genetic polymorphism surrounding differences in estrogen metabolism, as well as increased local uterine responsiveness to estrogen, has been implicated in Black and Asian individuals.^30,31,32^ This commonality could partially explain the higher prevalence seen in these groups. However, fibroid pattern in terms of size and multiplicity was significantly different between Black and Asian participants (Table 2), signaling that hormonal contribution is only a piece of the complex underlying mechanism. For instance, increased BMI has been associated with decreased sex hormone-binding globulin and hyperinsulinemia, which may result in a hormonal milieu that favors mitotic activity and fibroid growth.^33,34,35^ In our exploratory analysis, obesity was an independent risk factor for fibroids (eTable in Supplement 1). Yet, the high prevalence of uterine fibroids in Asian participants, who had the lowest BMI as a cohort in our study, supports the notion that fibroid growth is more than just hormonally mediated. Multiple endocrine-disrupting chemicals (EDCs), such as phthalates and parabens, have been implicated in fibroid growth.^36^ Prior studies found Black individuals to have higher levels of phthalates compared with White individuals, possibly secondary to differences in use pattern of personal care products.^37,38^ Phthalates may potentiate fibroid growth by delaying apoptosis and promoting leiomyoma cell survival.^39^ Higher urinary levels of paraben, an estrogenic preservative, were also observed in Black individuals.^40^ Limited data also show higher EDC levels in Chinese American female individuals.^41^ Furthermore, in in vitro studies, Vitamin D inhibits proliferation and extracellular matrix production in human leiomyoma cells.^42^ Deficient vitamin D levels, and their correlation with increased fibroid burden, have been observed in both Black and Chinese individuals.^43,44,45,46,47^ It is plausible that there exists a genetic predisposition to uterine fibroid development, which then undergoes variable progression based on individual exposures and risk factors influenced by different sociodemographic factors.

Higher fibroid burden, compounded by systemic inequity in quality of care, likely contribute to Black female patients experiencing advanced disease severity and facing an increased risk for surgically related complications. Health disparities in Asian patients with fibroids should also be investigated. We hope that our findings will promote adequate enrollment of highly affected groups in research studies to reduce health disparities associated with this pathology. Furthermore, both Black and Asian patients face worse outcomes after in vitro fertilization (IVF) treatment compared with White patients, including lower clinical pregnancy and live birth rates as well as higher rates of spontaneous abortion.^48,49,50^ The specific contribution of uterine fibroids to disparities in IVF treatment outcomes, and possibly adverse obstetrical outcomes, should be investigated given the commonality of increased fibroid burden in Black and Asian patients.

Strengths of this study include prospective examination of a large cohort of female participants and utilization of transvaginal ultrasonography to ascertain fibroid presence regardless of symptoms. All ultrasonographic examinations were performed by 2 board-certified reproductive endocrinologists with extensive experience in uterine assessment. The diverse and nonclinical nature of the cohort provided estimation of fibroid prevalence across different age and racial and ethnic groups. These data will be helpful for patient education and clinician awareness, which will in turn lead to timely diagnosis and indicated interventions.

### Limitations

This study has limitations. Excluding individuals with a history of pelvic surgery likely led to an underestimate of fibroid prevalence, as well as size and multiplicity, especially in older age groups. In addition, exclusion of individuals who had pelvic surgery or used hormonal medications may bias the ORs and predictive probabilities reported in our study, especially for at-risk groups. For instance, Black female individuals had earlier onset of and higher fibroid burden and were more likely to have undergone medical and/or surgical treatment. Therefore, estimates for Black participants (and possibly Asian participants) may be particularly vulnerable to underestimation. We also did not account for possible interobserver variability between the 2 examiners. However, the variability is likely of limited significance given the apparent nature of fibroid presence on transvaginal ultrasonography. Our cohort of Asian participants were all of Chinese descent, which may limit the generalizability of our findings to other Asian ethnicities. However, in another study evaluating a Canadian cohort with symptomatic fibroids, East Asian participants of various ethnicities, similar to Black participants, collectively had increased fibroid burden compared with White participants.^51^ East Asian participants also had a higher likelihood of being anemic in that study. We postulate that increased fibroid burden is not limited to Chinese individuals. Additional information on the fibroids, such as location and endometrial cavity distortion, was not provided given the limited capability of routine pelvic ultrasonography in fully determining these characteristics. Further investigation on the association between fibroid characteristics and reproductive outcomes would be invaluable for patient counseling and surgical decision making.

## Conclusion

In a community-based, diverse cohort of reproductive-age female individuals, 35.7% of Black or African American and 21.8% of Asian-Chinese participants were affected by fibroids, compared with 12.7% in Hispanic or Latina and 10.7% in White participants. Clinicians and patients should be aware of these differences to facilitate timely diagnosis and indicated treatments. Future studies should aim to identify specific fibroid characteristics that are associated with adverse reproductive outcomes in female individuals.

## Notes

Supplement 1. **eTable.** Logistic Regression Analyses of Association Between Race and Ethnicity and Presence of Fibroids

Supplement 2.
Data Sharing Statement
