# Supplementary material for: Presence of Fibroids on Transvaginal Ultrasonography in a Community-Based, Diverse Cohort of 996 Reproductive-Age Female Participants
Source: JAMA Netw Open. 2023 May 10;6(5):e2312701. doi: 10.1001/jamanetworkopen.2023.12701 (PMC10173016; doi:10.1001/jamanetworkopen.2023.12701)
Supplement: Supplement 1. — eTable. Logistic Regression Analyses of Association Between Race and Ethnicity and Presence of Fibroids [file jamanetwopen-e2312701-s001.pdf]

Supplemental Online Content

Huang D, Magaoay B, Rosen MP, Cedars MI. Presence of fibroids on transvaginal ultrasonography in a community-based, diverse cohort of 996 reproductive-age female participants. *JAMA Netw Open.* 2023;6(5):e2312701. doi:10.1001/jamanetworkopen.2023.12701

eTable. Logistic Regression Analyses of Association Between Race and Ethnicity and Presence of Fibroids

This supplemental material has been provided by the authors to give readers additional information about their work.

© 2023 Huang D et al. *JAMA Network Open.*

eTable. Logistic Regression Analyses of Association Between Race and Ethnicity and Presence of Fibroids

	Unadjusted OR		Adjusted OR Model 1[a]		Adjusted OR Model 2[b]	
**Variable**	**OR (95% CI)**	***P* value**	**OR (95% CI)**	***P* value**	**OR (95% CI)**	***P* value**
Race/ethnicity (compared to White)						
Black	4.65 (2.94-7.36)	<0.01	4.85 (3.01-7.81)	<0.01	4.72 (2.72-8.18)	<0.01
Hispanic	1.21 (0.71-2.08)	0.48	1.24 (0.72-2.15)	0.43	1.40 (0.74-2.64)	0.31
Asian	2.34 (1.43-3.82)	<0.01	2.50 (1.51-4.54)	<0.01	3.35 (1.95-5.76)	<0.01
Age (compared to 25-29)						
Age 30-34			1.75 (0.95-3.26)	0.08	1.80 (0.96-3.37)	0.07
Age 35-39			4.47 (2.55-7.82)	<0.01	5.01 (2.80-8.95)	<0.01
Age 40-45			5.65 (3.25-9.85)	<0.01	6.18 (3.46-11.05)	<0.01
Parous status					0.71 (0.48-1.06)	0.10
Current smoking					2.75 (1.55-4.89)	<0.01
Obesity (BMI ≥ 30)					1.63 (1.08-2.46)	0.02
College graduate					0.96 (0.65-1.44)	0.86

[a]Model 1: adjusted for age

[b]Model 2: adjusted for age, prior parity, current smoking, obesity (BMI≥30), and college graduate attainment

© 2023 Huang D et al. *JAMA Network Open.*
