# Supplementary material for: Presence of Fibroids on Transvaginal Ultrasonography in a Community-Based, Diverse Cohort of 996 Reproductive-Age Female Participants
Source: JAMA Netw Open. 2023 May 10;6(5):e2312701. doi: 10.1001/jamanetworkopen.2023.12701 (PMC10173016; doi:10.1001/jamanetworkopen.2023.12701)
Supplement: Supplement 2. — Data Sharing Statement [file jamanetwopen-e2312701-s002.pdf]

Data Sharing Statement

Huang. Presence of Fibroids on Transvaginal Ultrasonography in a Community-Based, Diverse Cohort of 996 Reproductive-Age Female Participants. *JAMA Netw Open*. Published May 10, 2023. doi:10.1001/jamanetworkopen.2023.12701

Data
Data available: Yes
Data types: Deidentified participant data
How to access data: David.Huang@ucsf.edu
When available: With publication

Supporting Documents
Document types: None

Additional Information
Who can access the data: Researchers whose proposed use of the data has been approved
Types of analyses: For a specified purpose and research question, or as baseline data for future studies
Mechanisms of data availability: With investigator support, after approval of a proposal, and with a signed data access agreement
Any additional restrictions: N/A
